# Can the benefits of cannabinoid receptor stimulation on neuroinflammation, neurogenesis and memory during normal aging be useful in AD prevention?

**DOI:** 10.1186/1742-2094-9-10

**Published:** 2012-01-16

**Authors:** Yannick Marchalant, Kevin Baranger, Gary L Wenk, Michel Khrestchatisky, Santiago Rivera

**Affiliations:** 1 Aix-Marseille Univ., NICN, UMR 7259, 13344, Marseille, France; 2 CNRS, NICN, UMR 7259, 13344, Marseille, France; 3 The Ohio State University, Psychology Department, Columbus, OH, USA

**Keywords:** Alzheimer's disease, Ageing, cannabinoids, neuroinflammation, neurogenesis, prevention

## Abstract

**Background:**

Alzheimer's disease has become a growing socio-economical concern in developing countries where increased life expectancy is leading to large aged populations. While curing Alzheimer's disease or stopping its progression does not appear within reach in a foreseeable future, new therapies capable of delaying the pathogenesis would represent major breakthroughs.

**Presentation of the hypothesis:**

The growing number of medical benefits of cannabinoids, such as their ability to regulate age-related processes like neuroinflammation, neurogenesis and memory, raise the question of their potential role as a preventive treatment of AD.

**Testing the hypothesis:**

To test this hypothesis, epidemiological studies on long term, chronic cannabinoid users could enlighten us on the potential benefits of these compounds in normal and pathological ageing processes. Systematic pharmacological (and thus more mechanistic) investigations using animal models of Alzheimer's disease that have been developed would also allow a thorough investigation of the benefits of cannabinoid pharmacotherapy in the pathogenesis of Alzheimer's disease.

**Implications of the hypothesis:**

The chronic administration of non-selective cannabinoids may delay the onset of cognitive deficits in AD patients; this will dramatically reduce the socio-economic burden of AD and improve the quality of life of the patients and their families.

## Background

Alzheimer's disease (AD) is the most common neurodegenerative disease and accounts for the majority of diagnosed dementia after age 60. Currently available drugs only produce temporary relief from some cognitive symptoms without delaying, stopping or reversing the neuropathology. Due to poor efficacy of current treatments and the likely delay to implement future safe and efficacious treatments, there is an opportunity to develop preventive approaches based on currently available knowledge regarding the pathogenesis of AD. Neuroinflammation has attracted growing attention due to its slow progression and chronic nature, particularly during normal aging, as well as its involvement in various neurodegenerative diseases [1]. Neuroinflammation has thus been targeted by numerous pharmacological agents [1-4]. Elderly patients (65 years and older) treated with non-steroidal anti-inflammatory drugs (NSAIDS) for 24 months exhibited a surprisingly lower prevalence of developing AD years later (follow up of 8 years, [5]). Subsequent clinical trials with NSAIDS were conducted on AD patients but failed to demonstrate significant beneficial effects [6]. Indeed, studies show that NSAIDS tend to lose efficacy in aged animals [7], which may account for the negative results of the clinical trials [6].

Most recent therapies have been designed and tested first in transgenic mice models and rarely take into account the ageing factor linked to AD development. As 95% of AD cases are non-familial and occur in the latter stages of life, basic knowledge on normal ageing processes and the way to delay them appear as a logical approach to tackle AD [8].

The endocannabinoid system has recently raised a great deal of interest in AD research, notably as a powerful modulator of neuronal activity (i.e. glutamatergic neurons) or inflammatory processes [9-11]. Cannabinoids already have numerous common uses as anti-emetics during cancer treatment or relief from inflammation-related pain [12]. Natural or synthetic cannabinoids exhibit variable specificity and selectivity for cannabinoid receptors [2]; this fact is particularly important in the light of the well-known psychoactive effect of some of these compounds due to their actions on neuronal CB1 receptors [13]. Despite the challenge of targeting receptors that potentially disrupt learning and memory, neuroprotective approaches have been taken to circumvent those effects by targeting more specifically the CB2 receptor, by modulating the degradation pathway of endocannabinoids, or by using low, non-psychoactive doses of non-selective agonists of CB1/CB2 receptors [14-16]. Indeed, in exploring the potential of cannabinoids in a preventive approach, we have recently demonstrated that non-psychoactive doses of a non-specific cannabinoid agonist (WIN-55,212-2) can decrease chronic neuroinflammation, restore hippocampal neurogenesis and improve memory in aged rats [17-20].

Hence cannabinoids are endowed with unique proclivities that warrant their use in the prevention of age-associated cognitive decline.

## Presentation of the hypothesis

From the above, we postulate that modulation of the endocannabinoid system in recently diagnosed AD patients by daily administration of low-doses of cannabinoids could at minimum delay the disease progression. In the long run, a preventive approach aimed at the general ageing population may become appropriate.

## Testing the hypothesis

Epidemiological studies have already been conducted on cannabis users, in particular in adolescents for the still controversial role of cannabinoids in the development of psychosis such as schizophrenia [21]. The popularity of cannabis among a significant number of people, notably in the 1970's, and the fact that some of these people still use cannabis chronically, could allow the identification of a cohort of chronic users who are currently over 60 years of age. If such a cohort could be constituted and cognitively tested, it would contribute to an evaluation of the effects of long term use of cannabinoids on brain ageing.

Further studies should be carried out on animals to address preclinical questions. These should involve chronic administration of different non-selective cannabinoids (e.g. WIN-55,212-2, cannabinol, Δ^9^-THC, HU-210, anandamide, or inhibitors of endocannabinoid degradation) in mouse models of AD. Indeed, mice have been genetically engineered to incorporate human mutations of genes encoding the amyloid precursor protein (APP) and presinilins (PS) linked to familial cases of AD, and/or the hyperphosphorylation of Tau protein [22-26]. Despite their "artificial" nature, transgenic mice represent a very convenient model to test various hypotheses in young and aged animals with reasonable efficiency in terms of time and costs. Among mouse models, hallmarks of AD pathology can be reproduced (amyloid plaques and/or neurofibrillary tangles, often associated with neuroinflammation) with an onset and progression depending on the "aggressivity" or number of mutations carried by the animals. These models would provide an interesting array of experimental conditions to test our hypothesis and notably the effects of cannabinoids on:

- Neuroinflammatory markers, now commonly associated with the development of the disease, such as glial cell responses and their inflammation-related production of specific inflammatory markers. Modulators of both inflammatory responses and APP/Aβ metabolism such as the metalloproteinases (ADAM-10, ADAM-17, MMP-2, -9, -14 and -25) remain to be explored.

- The expression of APP and its catabolites Aβ and sAPPα, as representatives of the amyloidogenic and non-amyloidogenic pathways, respectively. It will be of particular interest to evaluate the ratio of intracellular vs extracellular Aβ, and Aβ oligomers vs fibrils, considering the specificities of their toxicity.

- Tau protein hyperphosphorylation leading to formation of neurofibrillary tangles.

- Neurogenic processes that could slow or compensate ongoing neurodegeneration.

- Cognitive abilities tested using a variety of behavioral tasks.

## Implications of the hypothesis

The complex nature of AD advocates for the use of a multimodal drug approach that could protect from the various processes underlying neurodegeneration and thus, at minimum, delay the pathological process. The expected benefit from a chronic treatment aimed at stimulating the endocannabinoid system is a delayed progression of AD: i.e. reduced inflammation, sustained potential for neurogenesis, reduced accumulation of Aβ, reduced hyperphosphorylation of Tau and delayed memory impairment. Such results could lead to new therapeutic strategies that target both the chronic inflammation and the decline in neurogenesis associated with both normal ageing and AD. Most importantly, delaying disease progression will significantly reduce the number of severely impaired AD patients and thus reduce the growing socioeconomic burden associated with this disease.

## Competing interests

The authors declare that they have no competing interests.

## Authors' contributions

YM, KB, GLW, MK and SR participated to the conception of the present hypothesis. All authors drafted and approved the final manuscript.
